# Effect of metformin on inflammation and bone damage in a rat model of medication‐related osteonecrosis of the jaw

**DOI:** 10.1111/eos.70056

**Published:** 2025-12-04

**Authors:** Liviane Maria Alves Rabelo, Mariana Vasconcelos Guimarães, Aurilene Gomes Cajado, José Vitor Mota Lemos, Felipe Domingos de Sousa, Nylane Maria Nunes Alencar, Vilma de Lima, Paulo Goberlânio de Barros Silva, Ana Paula Negreiros Nunes Alves, Deysi Viviana Tenazoa Wong, Roberto César Pereira Lima‐Júnior

**Affiliations:** ^1^ Drug Research and Development Center Department of Physiology and Pharmacology Faculty of Medicine Federal University of Ceara Fortaleza Brazil; ^2^ Department of Dental Clinic Faculty of Pharmacy Dentistry and Nursing Federal University of Ceara Fortaleza Brazil; ^3^ Northeast Network of Biotechnology (RENORBIO) Centre for Experimental Biology (Nubex) University of Fortaleza (UNIFOR) Fortaleza Brazil

**Keywords:** bone healing, cell viability, interleukin‐1β, macrophage, zoledronic acid

## Abstract

This study investigated how chronic metformin administration modulates the cellular profile and inflammatory markers in a zoledronic acid‐based rat model of medication‐related osteonecrosis of the jaw (MRONJ). Male Wistar rats were allocated to different treatments: (i) naïve, (ii) MRONJ (zoledronic acid, 0.2 mg/kg, i.v. on days 0, 7, 14, and 49), or (iii) MRONJ + metformin (250 mg/kg, by gavage, daily for 70 days). All rats had the inferior first molar extracted on day 42. Mandibular arches were harvested for analyzing their gums on day 70. Additionally, RAW 264.7 cells were incubated with zoledronic acid or metformin for cell viability tests and analysis of interleukin‐1β (IL‐1β) production. MRONJ was characterized by increased numbers of empty osteocyte lacunae, osteoclasts, and apoptotic osteoclasts, and by high expression of tartrate‐resistant acid phosphatase (TRAP) and F4/80 (a macrophage marker). Zoledronic acid‐incubated RAW 264.7 macrophages showed increased IL‐1β expression. Metformin reduced the number of empty bone lacunae, apoptotic osteoclasts, leukocyte infiltrate, and F4/80 positive cells in the alveolar bone. It increased TRAP expression levels without altering the number of osteoclasts. Metformin also reduced the myeloperoxidase activity and decreased IL‐1β levels in vitro. In conclusion, metformin reduced the severity of MRONJ by mitigating the inflammatory response.

## INTRODUCTION

Bisphosphonates are drugs used to reduce bone resorption in osteoporosis in postmenopausal women and to prevent bony metastases in cancer cases [1, 2]. In addition to their antiresorptive efficacy, bisphosphonates are associated with two major adverse events, medication‐related osteonecrosis of the jaw (MRONJ) and atypical femoral fracture [3].

MRONJ is characterized by pain, infection, and a substantial reduction in quality of life. Its incidence depends on the therapeutic context, with a risk estimated at ≤0.05% for those taking oral bisphosphonates for osteoporosis and below 5% for patients receiving intravenous bisphosphonates or denosumab for bone metastases, though it has been reported to range up to 18% with prolonged therapy [4].

MRONJ's clinical management includes improved oral hygiene with regular professional dental maintenance, daily antimicrobial mouthrinses, oral antibiotics, and pain medication as needed when infection is present, and debridement of necrotic tissue as needed [5]. Tissue lesions are marked by necrosis, empty osteocyte lacunae, and the accumulation of lymphocytes and macrophages [6]. The bisphosphonate zoledronic acid is reported to increase peri‐implant osteogenesis via adenosine monophosphate‐activated protein kinase (AMPK) [7], which is the molecular target of metformin.

Metformin is one of the most prescribed drugs worldwide, being the first line of pharmacological treatment against type 2 diabetes mellitus [8]. Metformin has also shown promising anti‐inflammatory effects, including blockade of nuclear transcription factor‐κB and reduced secretion of pro‐inflammatory mediators [9]. A few reports describe the impact of metformin directly on bone tissue. Metformin decreases the expression of the NOD‐like receptor family pyrin domain‐containing 3 (NLRP3) inflammasome and cytokines such as interleukin‐1β (IL‐1β) [10]. Furthermore, metformin has been reported to prevent morphological alterations in the jawbones of rats with zoledronic acid plus dexamethasone‐induced osteonecrosis [11], but the mechanism was not investigated.

While the therapeutic potential of metformin for MRONJ has recently been explored in a model combining zoledronic acid and corticosteroids [11], the novelty of the present study lies in a clinically grounded animal model. Corticosteroids are, per se, risk factors and may confound the underlying zoledronic acid‐related pathogenesis by altering the inflammatory response [1, 12]. Therefore, to better isolate the effect of bisphosphonates and more accurately mimic the clinical scenario, we employed a zoledronic acid model. This approach allows us to specifically evaluate metformin's efficacy in preventing MRONJ in a pathophysiologically purer model, thereby strengthening the evidence for its therapeutic applicability.

The present study evaluated the cell profile and expression of osteoclastic and inflammatory markers during chronic metformin administration in an experimental model of zoledronic acid‐related osteonecrosis of the jaw.

## MATERIAL AND METHODS

### Animals

A total of 28 male Wistar rats (180–240 g, 8–9 months old) were used for the study. The central vivarium of the Federal University of Ceará provided the animals. They were kept in appropriate cages in a temperature‐controlled environment (22°C–25°C), with a relative humidity of 50%–60%, and under a 12‐h light/dark cycle. The animals also had free access to drinking water and standard food (Nuvilab). The Ethics Committee on the Use of Animals approved the study (approval number: 5,649,020,519), and all procedures were conducted in compliance with the animal research: reporting of in vivo experiments (ARRIVE) guidelines.

### Sample size calculation

**FIGURE 1:**
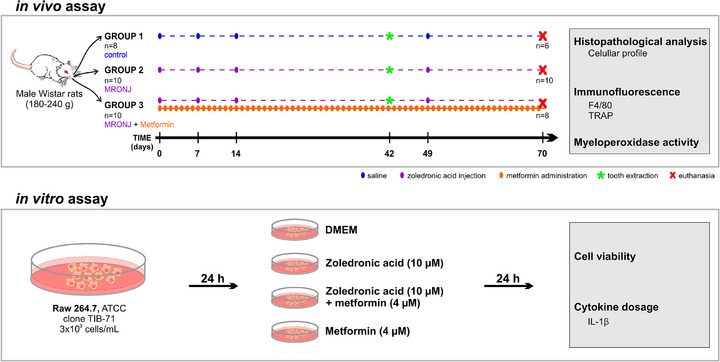
Experimental design and methodological overview of in vivo and in vitro analyses. In vivo assay timeline: male Wistar rats (*n* = 28) were allocated to one of three experimental groups (naïve control, *n* = 8; medication‐related osteonecrosis of the jaw [MRONJ], *n* = 10; MRONJ + Metformin, *n* = 10). The MRONJ groups received intravenous zoledronic acid (0.2 mg/kg) weekly (days 0, 7, 14, 49; purple dots), while the MRONJ + Metformin group also received daily oral metformin (orange dots). All animals underwent tooth extraction (green asterisk) on day 42 (two animals from the naïve and MRONJ + Metformin groups were excluded due to tooth fracture). Euthanasia occurred on day 70. Mandibles and gingival tissue were collected for histopathological analysis (cellular profile, immunofluorescence for F4/80 and tartrate‐resistant acid phosphatase [TRAP]) and myeloperoxidase activity assessment. In vitro assay timeline: RAW 264.7 macrophages were treated for 24 h with Dulbecco's modified Eagle's minimum essential medium (DMEM), zoledronic acid (10 µM), metformin (4 mM), or their combination. Cell viability and interleukin‐1β (IL‐1β) secretion were measured.

The sample size was determined a priori based on the primary outcome (empty bone lacunae/mm^2^). Using an estimated large effect size (Epsilon‐squared, *ε* ^2^ > 0.4) derived from previous MRONJ models [6, 11], a minimum of five animals per group was required to achieve 80% power at *α* = 0.05. To ensure robustness against predefined exclusion criteria (i.e., random mortality and tooth fractures during extraction), a total of 28 animals were initially allocated, resulting in group sizes of 8–10. This conservative approach also considered potential technical losses in histology and the unknown effect size of metformin treatment. Due to the 70‐day experimental timeline, no animal replacements were performed after exclusions were made to maintain temporal synchrony across all groups. Following exclusions (two naïve and two MRONJ + metformin animals due to tooth fractures), the final group sizes (*n* = 6–10, Figure 1) remained sufficient to provide robust power, thereby minimizing the risk of inconclusive results and adhering to the ethical principle of reduction by preventing the need for repeat experiments.

### Experimental groups

The animals were separated into three different experimental groups: naïve, MRONJ, and MRONJ + metformin, as detailed below and summarized in Figure 1.

*Control group (naïve, n = 8)*: These control animals were not subjected to MRONJ induction. They received 0.9% saline solution (5 mL/kg, i.v., on days 0, 7, 14, and 49 to mimic zoledronic acid injection). Additionally, the rats received the same solution by gavage (5 mL/kg, once a day) to simulate metformin administration. Furthermore, the lower left first molar was extracted on day 42 of the experiment.

For tooth extraction, the animals were previously anesthetized with ketamine/xylazine (90/10 mg/kg, i.p.). Syndesmotomy and dislocation procedures were performed with a number 05 exploration probe and a 3S spatula. The lower left first molar was then extracted with the aid of a Lecron spatula or Mayo needle holder. In this group, two animals with tooth fractures consequent to the extraction procedure were excluded from the study.

*MRONJ group (n = 10)*: The dose of 0.2 mg/kg was selected to model the clinical condition of MRONJ; the complete translational rationale is provided in the Discussion. This study followed the experimental model proposed by Silva and colleagues for MRONJ induction [6]. Zoledronic acid (SUN Pharma) was dissolved in sterile 0.9% saline solution. It was administered intravenously (0.2 mg/kg) once a week for three consecutive weeks (days 0, 7, and 14) through the dorsal penile vein. On experimental day 42, after the first zoledronic acid injection, the lower left first molar was extracted, as described previously. Animals with tooth fractures were excluded. One week after tooth extraction, corresponding to experimental day 49 after the first zoledronic acid administration, the animals received an additional injection of zoledronic acid (0.2 mg/kg, i.v.).

*MRONJ + metformin group (n = 10)*: Metformin (500 mg generic drug tablets, Merck) was dissolved in 0.9% saline solution and administered once a day (250 mg of active ingredient/5 mL/kg) by gavage from experimental day 0–70. Fresh solutions were prepared for each experimental day. The animals were also submitted to first molar extraction on day 42, as described previously, and received zoledronic acid as described in the MRONJ group. The metformin dose was chosen based on a previous study [11]. Two animals with tooth fractures were excluded.

### Obtaining samples after animal euthanasia

On experimental day 70, the animal euthanasia procedure consisted of the injection of a ketamine/xylazine solution (90/10 mg/kg, i.p.) followed by carotid exsanguination. The left mandibular hemiarchs and their respective gingival tissue adjacent to the area of interest (lower left first molar) were then removed from each animal and stored in 4% buffered formalin (the mandibular hemiarchs) or at −80°C (the gingival tissue).

### Histological processing of the mandibular hemiarchs

Microscopic analyses were conducted on tissue sections of the mandibular hemiarchs. The extracted hemiarchs were fixed in a 4% buffered formalin solution for 24 h. Following the fixation period, the specimens were subjected to a 25‐day demineralization using a 10% ethylene–diamine–tetraacetic acid buffered with 0.1 M phosphate solution to maintain a pH of 7.0. Tissue sections (4‐µm thick) in the sagittal plane were obtained to allow the visualization of the distal side of the third molar, the residual socket, and the mesial side of the first molar. The tissue sample was stained using the hematoxylin and eosin (H&E) method. The region of the residual socket related to the extraction of the lower left first molar was considered for the analysis.

### Histological evaluation: Cellular profile analysis

One tissue section, as equidistant as possible to the lingual and buccal sides of the residual socket, was evaluated per animal. H&E‐stained specimens were analyzed by light microscopy and photographed at a high‐magnification microscopic field (400×). To minimize variation in the quantity of mineralized bone tissue among the photographed areas under analysis, the complete slide was examined to count the total number of empty osteocyte lacunae, osteoclasts (viable and apoptotic), neutrophils, and mononuclear cells. The analysis was conducted using imagej software (US National Institutes of Health) [13]. Osteoclasts were considered apoptotic when exhibiting increased size, cytoplasmic vacuolization, and pyknotic nuclei, indicating nuclear chromatin condensation. The investigator (M.V.G.) responsible for cell counting was blinded to the treatment group from which the sections were obtained. A single, representative histological section from the region of interest was analyzed for each animal. The data presented for each group are the median and interquartile range of cell counts per animal.

### Expression of F4/80 and tartrate‐resistant acid phosphatase by immunofluorescence

The same blocks used for the histological analysis were cut into sections (4 µm) on poly‐L‐lysine‐covered slides (Dako Denmark Flex IHC microscope slides, Agilent Technologies Brasil) for the immunofluorescence analysis as described elsewhere [14]. Primary antibodies, specific to the target antigens, were as follows: rabbit anti‐F4/80 (a macrophage marker, diluted 1:200, product number D2S9R, Cell Signaling Technology) and polyclonal rabbit anti‐TRAP (tartrate‐resistant acid phosphatase, a marker expressed by osteoclasts and macrophages, diluted 1:100, product number PA5116970, Thermo Scientific). The antibodies were applied and incubated overnight at 4°C. After washing with phosphate‐buffered saline, an appropriate Alexa Fluor 568‐conjugated donkey anti‐rabbit secondary antibody (1:200, Invitrogen, Life Technologies, Thermo Fisher Scientific) was added and incubated for 2 h. 4′,6‐diamidino‐2‐phenylindole (DAPI) staining (4 µL in 200 mL of phosphate‐buffered saline, Invitrogen, Life Technologies, Thermo Fisher Scientific) for 30 min was employed to visualize nuclei. Image acquisition and analysis were performed using dedicated software (imagej, National Institutes of Health).

The fluorescent area was quantified blindly (A.G.C. was responsible for the analysis) by differentiating the red‐fluorescent pixels (Alexa Fluor 568) from the blue‐stained pixels used to identify DAPI‐labeled cell nuclei. Detection color thresholds were established and standardized in all quantifications. The data obtained were expressed as fluorescent areas by comparing the fluorescence intensity of the target marker with DAPI (100%).

### Assessment of myeloperoxidase activity in the gingival tissue

Myeloperoxidase activity in the gingival tissue surrounding the extraction area in the mandibular specimens was evaluated as described elsewhere [14]. The samples were incubated in an NaCl and disodium ethylene–diamine–tetraacetic acid solution diluted in phosphate buffer (0.02 M). The tissues were homogenized in a Qiagen TissueLyser LT (QIAGEN Biotecnologia Brasil) using 4.5‐mm steel beads. Myeloperoxidase activity was determined using a 1:4 mixture of tetramethylbenzidine (1.6 mM) and hydrogen peroxide (0.5 mM). The chemical reaction was halted using a 2 M sulfuric acid solution. The sample was analyzed using a spectrophotometer at 450 nm. The results were expressed as myeloperoxidase activity (neutrophils/mg of tissue) [15].

### In vitro culture of the murine macrophage cell line Raw 264.7

The murine macrophage cell line (Raw 264.7, ATCC clone TIB‐71) was purchased from the Rio de Janeiro Cell Bank and cultivated in Dulbecco's modified Eagle's minimum essential medium (DMEM) supplemented with 10% fetal bovine serum (Gibco Thermo Fisher Scientific) and 1% antibiotic (100 IU/mL penicillin/streptomycin 100 µg/mL, Sigma‐Aldrich) in an incubator at 37°C with 95% humidity and 5% CO_2_ atmosphere. Cell growth was observed under an inverted phase‐contrast microscope. Cell viability and IL‐1β levels were determined. The in vitro assay data comprised four independent biological replicates, each with three technical replicates. Experimental design and analytical overview are presented in Figure 1.

### Cell viability assay

The Alamar Blue method evaluated cell viability in Raw 264.7 cells (3 × 10^3^ cells/mL seeded in 96‐well plates) treated with zoledronic acid and metformin. After 24 h of cell growth, different concentrations of zoledronic acid (10 µM) or metformin (4 mM) were added to the wells (1.000–0.001 µg/mL in phosphate‐buffered saline) for 24 h. The control group received only DMEM. After the incubation period, the culture medium was removed, and a resazurin dye solution (0.312 mg/mL; VETEC) was added. The mixture was then incubated for an hour at 37°C and 5% CO_2_. Following this, the plates were analyzed using a microplate reader (BioTek Synergy HT) with an excitation wavelength of 530–560 nm and emission at 590 nm. The results of the viability test were expressed as a percentage of viable cells: viability (%) = [sample absorbance/mean absorbance of the DMEM control group] x 100.

### Cytokine dosage

The concentration of IL‐1β in the culture supernatant of Raw 264.7 macrophages was quantified using a commercial enzyme‐linked immunosorbent assay (ELISA) kit, according to the manufacturer's instructions (mouse IL‐1 beta DuoSet ELISA, R&D Systems) [16]. Supernatants were collected from cells treated for 24 h with DMEM (control), zoledronic acid (10 µM), metformin (4 mM), or zoledronic acid + metformin (10 µM and 4 mM, respectively), and stored at −80°C until analysis [16]. The reading was performed on a spectrophotometer at 450 nm. The results were plotted in a standard curve and expressed in pg/mL.

### Statistical analysis

After running the Shapiro–Wilk normality test, which showed significant deviation from normal distribution for all variables, the data were analyzed using the Kruskal–Wallis test and Dunn's post hoc test with Bonferroni correction for multiple comparisons. Results are presented as median with interquartile range to accurately reflect the non‐normal distribution and high variability observed in the data. All data points from surviving animals were included in the analysis, and no outliers were excluded. The analyses were performed using GraphPad Prism version 9.0 (GraphPad Software). A *p*‐value of less than 0.05 was considered statistically significant.

## RESULTS

### Analysis of bone parameters in the socket after tooth extraction

**FIGURE 2:**
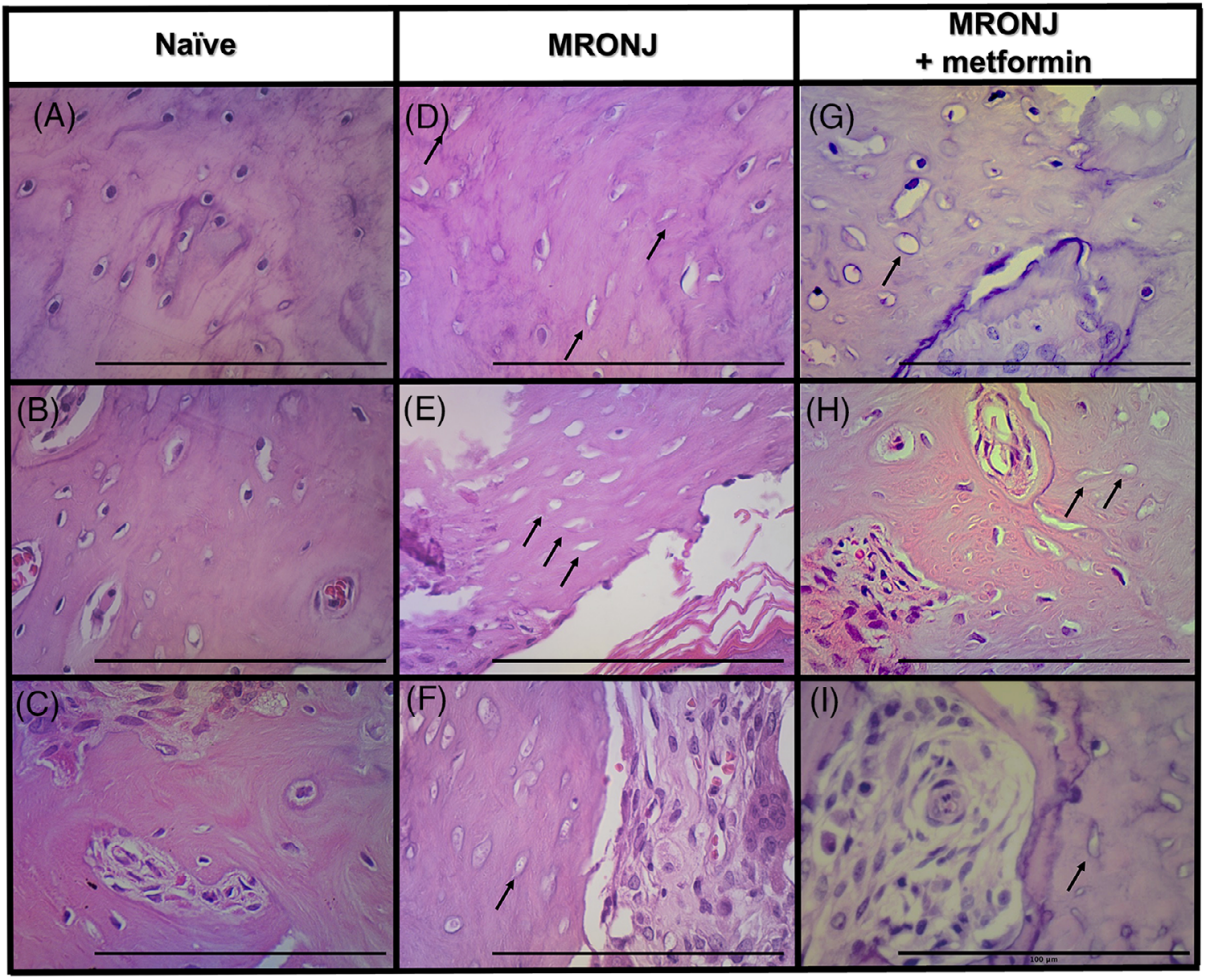
Representative photomicrographs of hematoxylin and eosin (H&E)‐stained tissues. The region analyzed was the bone tissue surrounding the healing socket. Photomicrographs of bone tissue represent images from three biologically independent animals in each group: naïve (A–C), medication‐related osteonecrosis of the jaw (MRONJ) (D–F), and MRONJ + metformin (G–I) rats. The black arrows denote areas of bone necrosis with empty osteocyte lacunae. The metformin‐treated group shows fewer empty lacunae than the zoledronic acid‐injected animals. (scale bar = 100 µm, magnification 400×).

**FIGURE 3:**
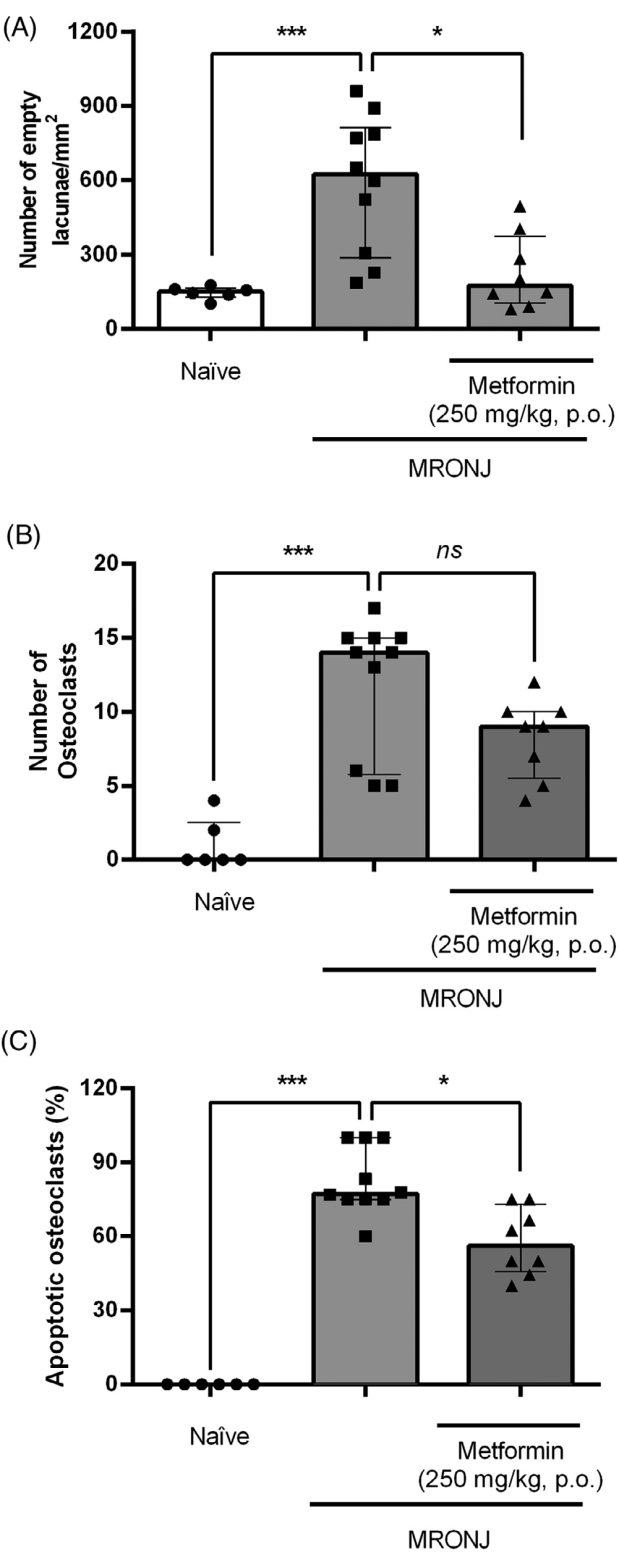
Cellular profile in the bone tissue. Male rats were divided into groups: naïve (*n* = 6), medication‐related osteonecrosis of the jaw (MRONJ) (*n* = 10), and MRONJ + metformin (*n* = 8). The region analyzed was the bone tissue surrounding the socket healing post‐extraction. The number of empty lacunae/mm^2^ of tissue is denoted in (A), the total number of osteoclasts (B), and the percentage of apoptotic osteoclasts (C). The data are presented as medians with interquartile ranges, and the differences between groups were analyzed using the Kruskal–Wallis test and Dunn post hoc test. *p < 0.05 and ***p < 0.001 represent a statistically significant difference between the groups under analysis; ns means no statistically significant difference.

Significant histological changes, marked by empty osteocyte lacunae suggestive of bone necrosis, were observed in animals injected with zoledronic acid (Figure 2D–F), compared with the naïve group, which demonstrated a typical osteocyte phenotype (Figure 2A–C). Quantification of the number of empty lacunae per mm^2^ confirmed a significantly higher number of osteocytes in the MRONJ group compared with the naïve control group (Figure 3A). Additionally, metformin administration attenuated bone necrosis compared with the MRONJ group (Figures 2G–I and 3A).

The zoledronic acid‐induced lesion was characterized by a significantly higher number of osteoclasts at alveolar sites in the MRONJ group than in the naïve group (Figure 3B). Such a finding was derived from the higher percentage of apoptotic osteoclasts in specimens from animals subjected to MRONJ than in naïve animals (Figure 3C).

Metformin administration did not significantly affect the total number of osteoclasts, which was similar to that seen in the MRONJ group (Figure 3B). However, the percentage of apoptotic osteoclasts was lower than seen in rats submitted to the MRONJ protocol (Figure 3C).

**FIGURE 4:**
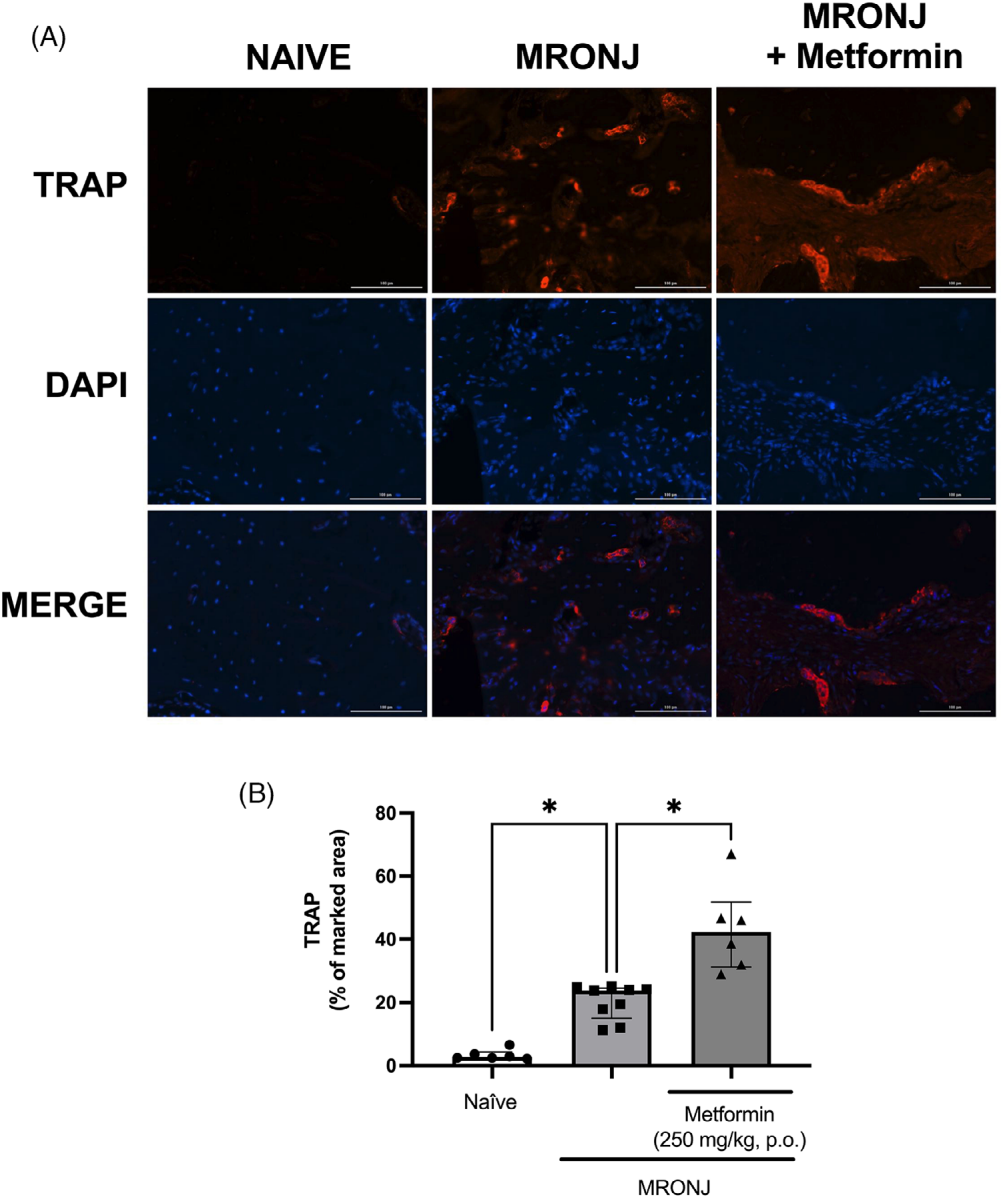
Tartrate‐resistant acid phosphatase (TRAP) expression by immunofluorescence. Representative images denoting the TRAP expression (A). The quantification is presented in (B). The region analyzed was the bone tissue surrounding the healing socket. The data are presented as medians with interquartile ranges, and the differences between groups were analyzed using the Kruskal–Wallis test and Dunn post hoc test. *p < 0.05 represents statistically significant differences between the groups under analysis. (scale bar = 100 µm, magnification 200×). The sample sizes vary between groups due to the loss of some tissue sections during staining. MRONJ, medication‐related osteonecrosis of the jaw; DAPI, 4′,6‐diamidino‐2‐phenylindole. MERGE: overlay image combining the fluorescent channels (red for TRAP, blue for DAPI) to visualize colocalization of markers with cell nuclei.

Osteoclast activity was assessed by immunofluorescence analysis of TRAP marker expression in the identical specimens submitted for H&E analysis. The administration of zoledronic acid resulted in higher expression of TRAP in the bone tissues (20.3% of median fluorescence intensity) than seen in the naïve group (3.4% of median fluorescence intensity). Additionally, animals with MRONJ treated daily with metformin showed a significantly higher in TRAP expression (43.2% of median fluorescence intensity) than animals in the MRONJ group (Figure 4A,B).

### Analysis of inflammatory parameters in the socket after tooth extraction

**FIGURE 5:**
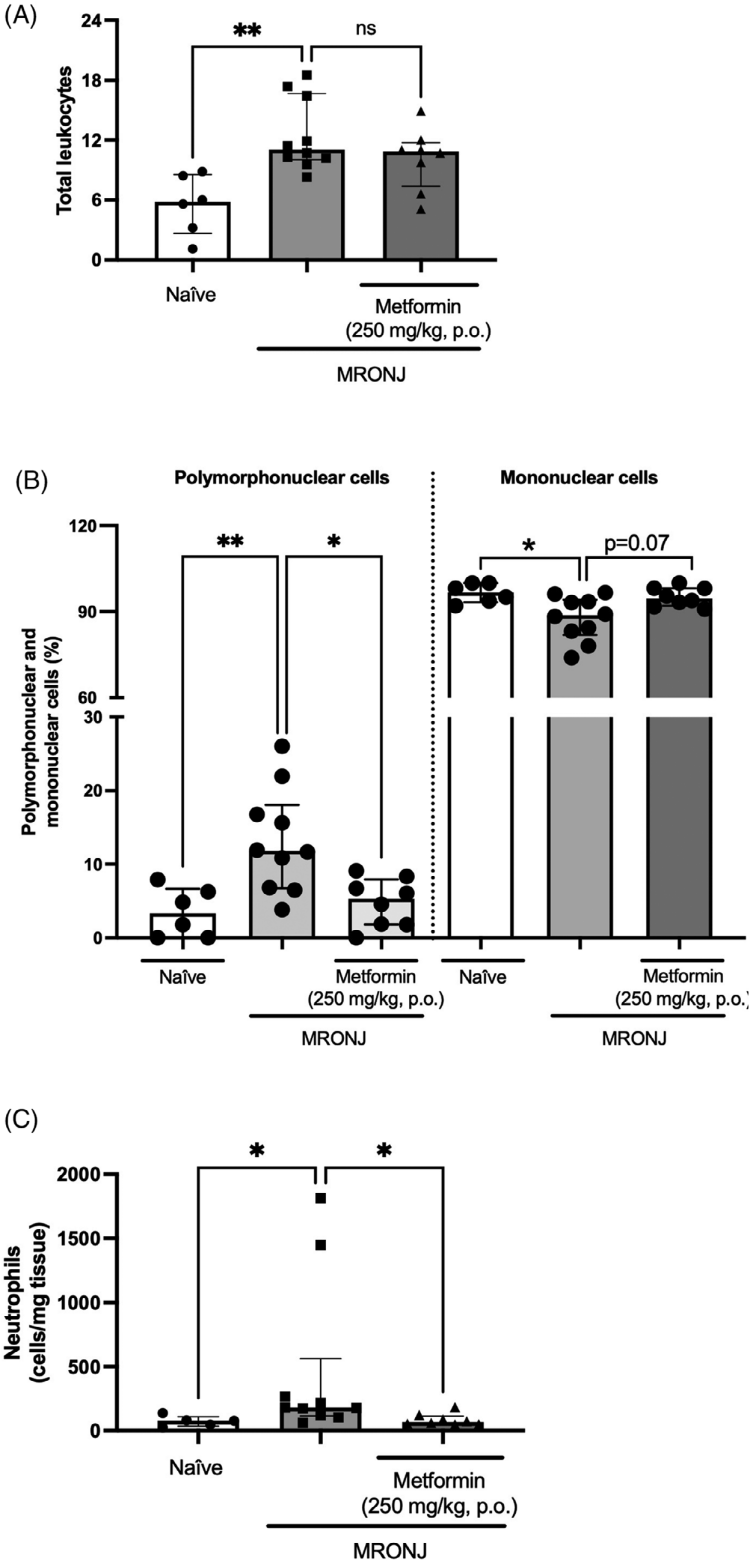
Effect of metformin administration on inflammatory parameters in animals with medication‐related osteonecrosis of the jaw (MRONJ). Treatment with metformin does not change the total number of leukocytes (A), but alters the percentage of polymorphonuclear cells (B) in the bone tissue surrounding the healing socket. Metformin also reduces neutrophil accumulation in the gingival tissue (C). The data are presented as medians with interquartile ranges, and the differences between groups were analyzed using the Kruskal–Wallis test and Dunn post hoc test. *p < 0.05 and **p < 0.01 flags statistically significant differences between the groups under analysis; ns means no statistical significance.

The induction of MRONJ augmented the total number of leukocytes in the alveolar bone tissue (11.5 [8.3–18.5] cells), which contrasted with the naïve group (5.8 [1.1–8.9] cells). Daily administration of metformin did not prevent the increase in total leukocytes caused by zoledronic acid (10.8 [5.1–14.9] cells) (Figure 5A).

The analysis of the polymorphonuclear and mononuclear percentages per group indicated that zoledronic acid altered the proportions of both cell types (11.8%:88.8%) compared with the naïve group (3.3%:96.7%). Notably, metformin administration maintained the respective percentages of polymorphonuclear:mononuclear cells similar to those of naïve animals (5.3%:94.7%), which were significantly different from those of the MRONJ group (Figure 5B).

The metformin anti‐inflammatory effect was also analyzed in the gingival tissue surrounding the tooth extraction site and assessed using myeloperoxidase activity to detect neutrophil infiltration. Zoledronic acid injection resulted in significantly higher neutrophil accumulation in the gingiva by 624% than seen in the naïve group. In contrast, daily metformin treatment attenuated neutrophil infiltration by 96% compared with the MRONJ group (Figure 5C).

**FIGURE 6:**
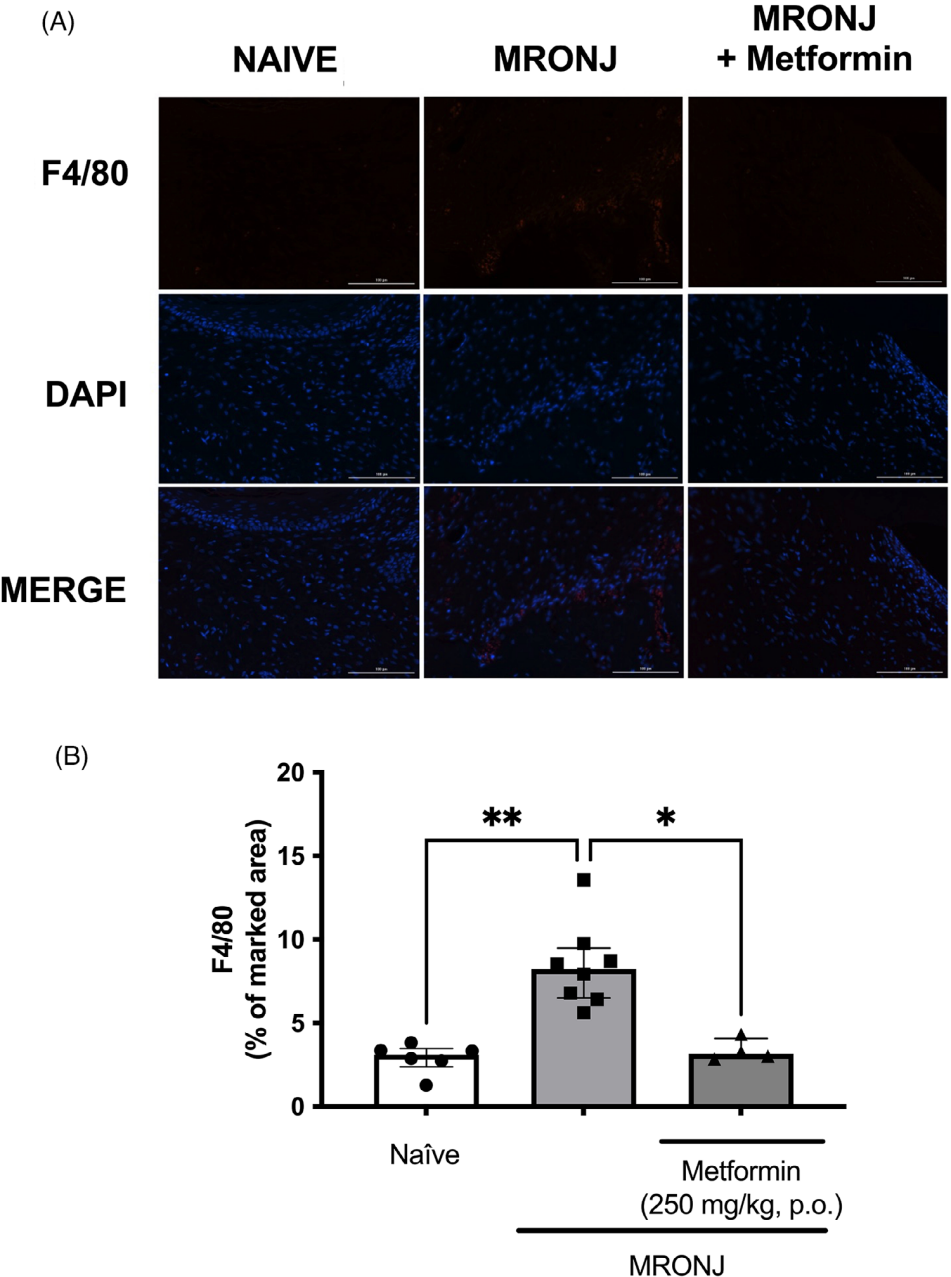
F4/80 expression by immunofluorescence. The region analyzed was the bone tissue surrounding the healing socket. Representative images denoting the F4/80 expression in the experimental groups (A). The quantification of the fluorescent area (%) is shown in (B). The data are presented as medians with interquartile ranges, and the differences between groups were analyzed using the Kruskal–Wallis test and Dunn post hoc test. *p < 0.05 and **p < 0.01 represent statistically significant differences between the groups under analysis. (scale bar = 100 µm, magnification 200×). The sample sizes vary between groups due to the loss of some tissue sections during staining. MRONJ, medication‐related osteonecrosis of the jaw; DAPI, 4′,6‐diamidino‐2‐phenylindole. MERGE: overlay image combining the fluorescent channels (red for F4/80, blue for DAPI) to visualize colocalization of markers with cell nuclei.

The presence of F4/80 positive cells to denote a macrophage infiltration in the inflamed tissues was analyzed by immunofluorescence in the alveolar bone tissue post‐tooth extraction. The MRONJ group presented with higher F4/80 expression (8.2% of median fluorescence intensity) than the naïve control group (3.1% of median fluorescence intensity). Metformin treatment resulted in an F4/80 expression (3.1% of median fluorescence intensity), which was significantly lower than seen in zoledronic acid‐injected rats (Figure 6A,B).

### Effect of metformin on IL‐1β production by zoledronic acid‐stimulated macrophages

**FIGURE 7:**
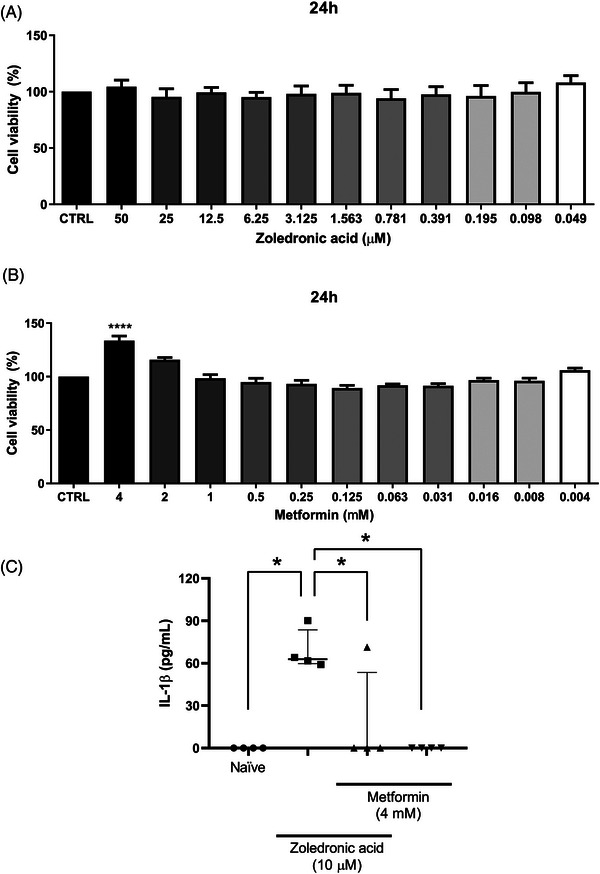
Effects of metformin on RAW 2647 cell viability and Interleukin‐1β (IL‐1β) levels. Cell viability was analyzed under various concentrations of zoledronic acid (A) and metformin (B). IL‐1β concentration in the cell supernatant was evaluated by enzyme‐linked immunosorbent assay (ELISA) after incubation with DMEM medium (Naive), zoledronic acid (10 µM) or metformin (4 mM) (C). The data represent the analysis of four independent experiments (biological replicates), each composed of three technical replicates. The data are presented as medians with interquartile ranges, and the differences between groups were analyzed using the Kruskal–Wallis test and Dunn post hoc test. *p < 0.05 represents statistically significant differences between the groups under analysis. **** *p* < 0.0001 versus DMEM control (CTRL) group.

Figure 7A shows that during the 24‐h incubation, zoledronic acid did not reduce the viability of Raw 264.7 at any of the tested concentrations. Additionally, incubation of macrophages with metformin resulted in significant cell growth at 4 mM (Figure 7B).

Analysis of IL‐1β levels in the supernatant of Raw 264.7 cells indicated that zoledronic acid induced a significant increase in IL‐1β levels compared with the DMEM group. Conversely, metformin alone or in combination with zoledronic acid reduced IL‐1β levels (Figure 7C).

## DISCUSSION

The present study found that chronic metformin administration significantly attenuated the development of zoledronic acid‐induced MRONJ in rats. Metformin's effect was demonstrated by fewer number of empty osteocyte lacunae, a hallmark of osteonecrosis, fewer apoptotic osteoclasts, and suppression of inflammatory mechanisms, including macrophage infiltration (F4/80) and IL‐1β production. Additionally, metformin restored a healthier balance of bone remodeling, as evidenced by a significant increase in TRAP expression.

Zoledronic acid is a bisphosphonate widely used to prevent bone metastases in risk situations, such as breast cancer [17]. Its mechanism of action primarily involves inhibition of osteoclasts, thereby reducing bone resorption. However, this drug has been associated with MRONJ and atypical femoral fractures as the two main adverse events.

The choice of male rats for this study first aimed to eliminate the confounding influence of the female estrous cycle on bone metabolism and inflammation, ensuring a more stable baseline in the MRONJ model. Second, clinical data, while indicating that the higher prevalence in females is a reflection of the underlying diseases (e.g., osteoporosis and breast cancer) for which antiresorptives are prescribed [4], also identifies that the patient cohort with the highest proportional risk of developing MRONJ following dentoalveolar surgery is often males treated for cancer with high‐dose intravenous regimens [18]. Therefore, the use of male rats in the present study was intended to model this specific high‐risk clinical scenario rather than to imply a biological sex‐based susceptibility.

The intravenous use of zoledronic acid at a dose of 0.2 mg/kg on experimental days 0, 7, 14, and 49, combined with tooth excision on experimental day 42, induced areas of bone necrosis in the analyzed mandibular specimens. The formation of empty osteocyte lacunae was proportionally related to the quantification of necrotic bone in our study, since that finding is considered an essential criterion for determining MRONJ [6].

The dosage of zoledronic acid in rodent models is a matter of debate [19]. In the clinical setting of cancer, more frequent dosing of zoledronic acid and poor oral health are associated with higher rates of MRONJ [20]. The 3‐year cumulative incidence of that side effect in cancer patients is 2.8% [20]. Zoledronic acid is administered 4 mg intravenously per patient (∼0.05 mg/kg) at variable schedules. The human dose corresponds to 0.3 mg/kg in rats [21]. Considering this premise and previous studies [6], this study used zoledronic acid at a dose of 0.2 mg/kg to induce experimental osteonecrosis.

The absence of a group receiving zoledronic acid without tooth extraction in our study limits our ability to delineate the chemotherapeutic isolated effects on baseline inflammation in the jawbone. Remarkably, a previous study demonstrated that while zoledronic acid alone altered bone microarchitecture, the most pronounced radiographic signs of delayed healing and open wounds were observed primarily in the groups that underwent tooth extraction [22]. It supports the concept that local trauma is a critical cofactor in disease pathogenesis. Standard methods of extracting healthy teeth and administering zoledronic acid in rats have been criticized for failing to replicate clinical practice, in which tooth extraction is often performed due to dental infectious diseases [23]. Yan and colleagues proposed an association between MRONJ and surgical interventions in the setting of pathologic inflammatory conditions [23]. Conversely, we argue for the validity of the rat model used in the present study, as the animals received no prophylactic antibiotics. It means that an underlying infectious component is added to the model at the tooth extraction site.

In the present study, only one sagittal tissue section per animal was analyzed. Given the known heterogeneity of MRONJ lesions, obtaining one tissue section per animal is a limitation. Rough sectioning is a crucial step in histological sample preparation, removing the superficial layers of the paraffin block before thin‐sectioning for microscopic analysis. The region of interest of the residual socket consists of a narrow area. Therefore, collecting more than a few histological sections is challenging. However, the sections were representative of MRONJ lesions, as homogeneity between the samples was achieved under the controlled conditions of the experimental assays.

We also evaluated the effect of metformin, an antidiabetic drug with anti‐inflammatory and immunomodulatory potential [10], on the development of MRONJ. Metformin was administered daily by gavage since the oral administration route is considered standard for the chronic use of this drug [24]. Metformin treatment prevented the increase in empty osteocyte lacunae compared to the untreated MRONJ group. The present study evaluated the effect of oral metformin administration for 70 experimental days, initiated before the first zoledronic acid injection. Our findings agree with a previous study that tested metformin in a rat model of MRONJ induced by the combination of zoledronic acid (0.1 mg/kg) and corticosteroids, using a 6‐week experimental protocol [11]. Notwithstanding similar findings in tissue morphology, our MRONJ model, induced solely by zoledronic acid and tooth extraction, better mimics the clinical disease. Adding corticosteroids to the model might paradoxically influence the underlying pathogenesis of MRONJ and confound the analysis [1]. Glucocorticoids impair the production of inflammatory‐driving mediators. However, dexamethasone potentiates the number of apoptotic osteoclasts and the level of infection, suggesting that it negatively changes the course of zoledronic acid‐related MRONJ and leads to a different underlying pathogenesis [12]. Despite differences between the present model and that of Nakagawa et al. [11], metformin administration protected against bone lesions in both experimental conditions.

Metformin is considered an AMPK agonist [25]. The AMPK pathway positively regulates the expression of genes that favor osteoblastic activity [26], such as RUNX2 and osteocalcin, and increases bone alkaline phosphatase activity, a marker of osteoblast activity [27]. Additionally, metformin stimulates the vascular endothelial growth factor receptor signaling, contributing to angiogenesis and local microcirculation [28]. Such factors may be associated with metformin's protective effect, though this was not tested. While the present study did not include direct molecular measurements of AMPK in vivo, its role as a primary cellular target of metformin makes it a plausible contributor to the observed preservation of bone vitality.

The inhibitory effect of bisphosphonates on osteoclasts is well known. The number of osteoclasts on the bone surface after bisphosphonate administration tends to increase, and after a more extended period of drug exposure, the number of osteoclasts is reduced [13, 29]. In the present MRONJ experimental protocol, we observed a significant increase in osteoclasts following zoledronic acid exposure, suggesting an early disease course. Our results align with previous reports showing that MRONJ is characterized by an increase in osteoclast number [13, 29]. Such an increase may result from a compensatory response induced by bisphosphonate exposure, which triggers osteoclast apoptosis [29]. A significant increase in osteoclasts considered morphologically apoptotic, with larger, pyknotic nuclei, was consistent with previous reports [29].

As observed, metformin did not modify the total number of osteoclasts but significantly reduced the percentage of apoptotic osteoclasts. In vitro findings suggest that metformin induces osteoclast apoptosis by activating AMPK [30]. This might be associated with the concentration of metformin and dependent on the duration of drug exposure [30].

Analyzing TRAP expression is considered an efficient method of evaluating osteoclast activity. In agreement with this, we found that MRONJ was accompanied by a significant increase in the expression of that marker. Despite reports indicating that bisphosphonates reduce osteoclast responsiveness, as evidenced by reduced TRAP expression [29], our result is consistent with a previous finding of increased TRAP expression in mandibular specimens from animals treated with zoledronic acid [11]. Notably, metformin significantly increased TRAP expression in animals with MRONJ. This observation contrasts with previous reports that metformin does not alter TRAP expression in other models of MRONJ [11] and that it inhibits osteoclasts [30]. However, it aligns with the number of empty osteocyte lacunae detected and the osteoclasts counting, in which metformin reduced MRONJ injury and the percentage of apoptotic cells. An alternative data interpretation could include a potential shift in osteoclast differentiation or function. Metformin modulates the bone microenvironment, for instance, by decreasing the receptor activator of nuclear factor‐κB ligand/osteoprotegerin ratio [31], an essential mechanism of osteoclastogenesis regulation. The increased TRAP expression in our model may reflect a metformin‐driven, compensatory modulation of osteoclast activity within a complex MRONJ pathophysiology, rather than a straightforward inhibition. Additionally, it is suggested that the antiangiogenic effect of bisphosphonates impairs nutrient supply to the tissue and promotes the accumulation of toxic metabolites, directly influencing osteoclast death [32]. The positive effect of metformin on the angiogenic process in hypoxic or ischemic conditions [33], though not tested in this study, may also explain the improved bone parameters observed.

We observed that MRONJ was marked by polymorphonuclear and mononuclear cell infiltration. The involvement of inflammatory mediators in MRONJ development following zoledronic acid exposure prompted us to speculate on the anti‐inflammatory potential of metformin in this experimental model.

The complete pathogenic network of MRONJ remains to be described. Increased cytokine concentrations, such as tumor necrosis factor‐α, IL‐1β, and other inflammatory mediators, appear essential for the development of MRONJ [34]. It is known that these cytokines induce osteoclastogenesis and increase bone turnover [35]. In susceptible patients, including those treated with bisphosphonates, bone turnover demand, combined with osteoclast inhibition, fuels the development of osteonecrosis.

Notably, systemic administration of metformin did not change zoledronic acid‐associated total leukocyte accumulation in the bone healing socket, as detected by the H&E analysis. However, it reduced the F4/80 fluorescence compared with the MRONJ group. Such apparent divergent data might be due to differences in sensitivity between the methods used. It indicates that the immunofluorescence analysis offers superior sensitivity and signal amplification in this experimental model.

MRONJ is characterized by exposure of bone tissue and delayed wound healing. The deleterious effects of bisphosphonates on bone microcirculation also affect the inflammatory infiltrate in surrounding tissues [36]. Then, the effect of metformin on the gingival soft tissue surrounding the necrotic area was assessed by measuring neutrophil myeloperoxidase activity. MRONJ increased neutrophil infiltration in the gingiva, in accordance with a previous study in which a bisphosphonate increased myeloperoxidase activity in a culture of neutrophils obtained from human gingival crevicular fluid [37]. Remarkably, metformin reduced neutrophil accumulation in the surrounding tissue, as demonstrated in a previous study, suggesting its capacity to reduce oxidative stress, myeloperoxidase activity, and tissue damage in animal models of diabetes and prostate cancer [38].

Based on the in vivo data, we further validated the anti‐inflammatory effect of metformin in vitro by measuring IL‐1β levels in supernatants from RAW 264.7 macrophage cultures treated with zoledronic acid. Zoledronic acid and metformin were used at 10 µM and 4 mM, respectively, with an incubation period of 24 h. Such concentrations did not reduce cell viability.

In the present in vitro analysis, zoledronic acid treatment increased IL‐1β concentration in cell supernatant. Zoledronic acid has been reported to increase toll‐like receptor 4 expression in macrophages, favoring an M1 macrophage phenotype and the production of pro‐inflammatory cytokines, including IL‐1β [39]. Remarkably, MRONJ induction in diabetic animals has been reported to occur via an IL‐1β‐dependent mechanism [40]. Such studies suggest that the underlying MRONJ pathogenesis associated with zoledronic acid injection depends on the activation of the toll‐like receptor 4/IL‐1β pathway, thus corroborating our findings.

The mechanistic basis for metformin's protective effect, while not directly measured in our in vivo model, is supported by in vitro data and the established literature [41, 42]. According to the present findings, metformin treatment, per se, did not increase IL‐1β levels in RAW 264.7 cultures but prevented the production of that cytokine during zoledronic acid treatment. Since IL‐1β activation is a canonical process regulated by the NLRP3 inflammasome [41], our results suggest that metformin's benefit may be mediated, at least in part, through the suppression of this pathway. This interpretation is supported by a previous study demonstrating that metformin reduces NLRP3 inflammasome activity in macrophages under other pro‐inflammatory conditions [42].

Metformin may have reduced MRONJ injury, as evidenced by fewer empty osteocyte lacunae, at least in part through anti‐inflammatory mechanisms. The reduction in IL‐1β and F4/80 expression following metformin treatment has been reported in cancer models [43], indicating the importance of AMPK in metformin's anti‐inflammatory effects. Such effects may have reverberated in the drug's regulatory potential for bone parameters evaluated, alleviating the MRONJ injury induced by zoledronic acid.

An increase in osteoclast activity would be unsuitable for most bone conditions. However, in the specific context of MRONJ, there is an unbalanced pathologic shutdown of healthy bone turnover and a disruption of osteoclast‐osteoblast coupling. Collectively, our data suggest that metformin is not causing pathological bone resorption but is instead restoring a healthier bone remodeling balance, which is crucial for healing. Possible explanations include that zoledronic acid induces osteoclast apoptosis, leading to the formation of non‐viable, necrotic bone. It creates a pathological environment where bone cannot be repaired. Our key finding is that metformin specifically reduced the percentage of apoptotic osteoclasts without changing the total osteoclast number. Simultaneously, metformin significantly increased TRAP expression, a marker of osteoclast activity. It indicates that metformin may be rescuing osteoclasts from zoledronic acid‐induced apoptosis and promoting the activity of surviving cells. A third important point is that the beneficial effect on bone is likely secondary to metformin's anti‐inflammatory action, as evidenced by the reduction in IL‐1β and neutrophil infiltration. The pro‐inflammatory milieu in MRONJ is toxic to bone cells. Metformin creates a favorable environment for bone cell survival and function by mitigating the inflammatory response.

In summary, administering zoledronic acid to induce MRONJ at a dose equivalent to that used to treat cancer patients successfully caused significant bone and inflammatory changes in the post‐extraction healing socket and in the gingival tissue analyzed. Metformin treatment attenuated MRONJ, as confirmed by quantification of empty osteocytes and apoptotic cells, increased TRAP expression, and reduced F4/80 expression. MRONJ development affected the surrounding gingiva, as confirmed by augmented neutrophil accumulation, which metformin also attenuated. The metformin anti‐inflammatory effect was further confirmed in macrophages in in vitro conditions. These data support testing the anti‐inflammatory effect of metformin in the clinical setting to prevent the establishment of MRONJ.

## AUTHOR CONTRIBUTIONS

**Conceptualization**: Deysi Viviana Tenazoa Wong and Roberto César Pereira Lima‐Júnior. **Funding acquisition**: Nylane Maria Nunes Alencar and Roberto César Pereira Lima‐Júnior. **Investigation**: Liviane Maria Alves Rabelo, Mariana Vasconcelos Guimarães, Aurilene Gomes Cajado, José Vitor Mota Lemos, and Felipe Domingos de Sousa. **Methodology**: Felipe Domingos de Sousa, Deysi Viviana Tenazoa Wong, and Roberto César Pereira Lima‐Júnior. **Data curation**: Liviane Maria Alves Rabelo, Mariana Vasconcelos Guimarães, Aurilene Gomes Cajado, and José Vitor Mota Lemos. **Formal analysis**: Liviane Maria Alves Rabelo, Mariana Vasconcelos Guimarães, Aurilene Gomes Cajado, José Vitor Mota Lemos, Felipe Domingos de Sousa, Vilma de Lima, Paulo Goberlânio de Barros Silva, Ana Paula Negreiros Nunes Alves, and Roberto César Pereira Lima‐Júnior. **Writing—original draft**: Mariana Vasconcelos Guimarães and Roberto César Pereira Lima‐Júnior. **Writing—review and editing**: Liviane Maria Alves Rabelo, Mariana Vasconcelos Guimarães, Aurilene Gomes Cajado, José Vitor Mota Lemos, Felipe Domingos de Sousa, Nylane Maria Nunes Alencar, Vilma de Lima, Paulo Goberlânio de Barros Silva, Ana Paula Negreiros Nunes Alves, Deysi Viviana Tenazoa Wong, and Roberto César Pereira Lima‐Júnior.

## CONFLICT OF INTEREST STATEMENT

The authors declare no conflicts of interest.
